# Identification of hub genes and immune-related pathways in acute myeloid leukemia: insights from bioinformatics and experimental validation

**DOI:** 10.3389/fimmu.2024.1511824

**Published:** 2025-01-10

**Authors:** Mingliang Shan, Li Xu, Wenzhe Yang, Shiguo Liu, Zhaoqing Cui

**Affiliations:** ^1^ Postdoctoral Workstation, Liaocheng People’s Hospital, Liaocheng, China; ^2^ Postdoctoral Mobile Stations, The Affiliated Hospital of Qingdao University, Qingdao, China; ^3^ Post - Doctoral Innovation Practice Base, Gaomi Maternity and Child Health Hospital, Gaomi, China; ^4^ School of Management, Shandong Second Medical University, Weifang, China; ^5^ College of Acupuncture and Massage, Shandong University of Traditional Chinese Medicine, Jinan, China

**Keywords:** AML, hub gene, bioinformatics, machine learning, Mendelian randomization, plasmid, RT-qPCR

## Abstract

**Background:**

This study aims to identify the hub genes and immune-related pathways in acute myeloid leukemia (AML) to provide new theories for immunotherapy.

**Methods:**

We use bioinformatics methods to find and verify the hub gene. At the same time, we use the results of GSEA enrichment analysis to find immune-related mediators. Through Mendelian randomization(MR) analysis, on the one hand, we look for related immune cells, and on the other hand, we use it to determine the causal relationship among immune cells, immune mediators, and AML. Finally, *in vitro* experiments are conducted to further verify and improve the reliability and physiological functions of the hub gene and its immune-related pathways.

**Results:**

Complement Factor D(*CFD*) gene is identified as the highly expressed hub gene and is positively correlated with IL-2. IL-2 is also positively correlated with CD27 on CD24+CD27+B cells, JAK/STAT, and PI3K/Akt. The latter three are positively correlated with the occurrence and development of AML.

**Conclusion:**

We conclude that *CFD* gene uses IL-2 as a mediator to promote the disease progression of AML by promoting the CD27 on CD24+CD27+B cells, JAK-STAT, and PI3K-Akt pathways.

## 1 Introduction

AML is a common acute leukemia that can occur in individuals of all ages (1, 1). The diagnosis and treatment of AML often suffer from a lack of sensitive and specific biomarkers, with most patients diagnosed at intermediate or advanced stages (2). Furthermore, there are limited treatment options for AML, and it is common for drug resistance to develop (3, 4). Although treatment has been administered, the recurrence rate of patients with AML remains very high (5, 6), which leads to a very low overall survival rate (7, 8). Therefore, identifying hub genes and associated mechanisms for pathogenesis, proliferation, and recurrence is crucial for early screening, accurate diagnosis, effective treatment strategies, and prognosis assessment (9, 10).

The use of immune pathways to combat cancer cells has a history of a hundred years (11). The first successful clinical application of immunotherapy is the use of allogeneic hematopoietic stem cell transplantation (12). In recent years, immunotherapies like chimeric antigen receptor (CAR) have begun to gain people’s attention (3, 13, 14). Studies have shown that it is of crucial importance to improve the immune efficacy and reduce toxic and side effects through immune analysis related to AML (15–17). At present, there is still a great lack in this aspect (14, 18). Therefore, studying AML-related hub genes (19) and related immune pathways can provide valuable insights for AML in the aspect of immunotherapy (20).

In this study, our aim is to first screen for hub genes. Through the screening and validation of differential genes, the hub genes related to the disease can be identified. And the GSEA analysis of all the co-expressed genes of hub genes can discover the pathways they regulate. By this means, in this study, we can identify the inflammatory factors regulated by hub genes. The immune infiltration analysis is then used to further confirm the immune association between hub genes and the disease. Afterwards, bulk MR is utilized to screen for immune cells, and mediation MR is employed to determine the relationship among inflammatory factors, immune cells and AML. We can judge that there is a positive correlation among them through this method, which provides guidance for the subsequent *in vitro* validation. Finally, through *in vitro* experiments, we verify the reliability of hub genes and the impact of possible immune-related pathways on cell proliferation. We aim to reveal that hub genes affect and regulate the occurrence and development of AML through multiple immune-related pathways, providing new inspiration for improving the immunotherapy effect of AML.

## 2 Materials and methods

### 2.1 Bioinformatics analysis

#### 2.1.1 Data sources

The datasets were obtained from the GEO database (https://www.ncbi.nlm.nih.gov/geo/). We searched the GEO database using the keywords “acute myeloid leukemia “ [MeSH Terms] AND “Homo sapiens” [porgn: txid9606] and “Expression profiling by array” [All Fields]. The criteria for screening included the following: the microarray dataset referred to the genome-wide gene expression profiles in blood. The microarray dataset contained samples from AML and samples from healthy conditions. The included samples were not associated with any other diseases. The number of AML samples needed to be greater than 10. Based on the above conditions, we screened GSE9476 (including 38 normal samples and 26 AML samples) and GSE24395 (including 5 normal samples and 12 AML samples). These datasets were merged to form a new dataset which eliminated batch effects to form the experimental dataset. GSE30029 (comprising 31 normal and 90 AML samples) served as the validation dataset.

#### 2.1.2 DEGs selection and functional enrichment analysis

We used the “limma” package in R to identify DEGs, with a threshold of P < 0.05 and |log2 FC| > 2. Subsequent analyses included gene ontology (GO), Kyoto Encyclopedia of Genes and Genomes (KEGG) enrichment analyses, and immune-related gene set enrichment analysis (GSEA). Statistical significance was defined by a P value of 0.05, with a threshold of |log2 FC| > 1 applied for GSEA.

#### 2.1.3 Weighted gene co-expression network analysis

We clustered the samples and removed outliers. The optimal power value was determined to be 11, which was used to assess the fit index and average connectivity. Based on this optimal power value, a scale-free network was constructed. The efficacy of this construction was evaluated by plotting the topology of the scale-free network, which allowed for the generation of a distance matrix for gene clustering. Subsequently, dynamic module identification was conducted, focusing on modules containing at least 30 genes. Highly correlated modules were clustered and merged. Heatmaps illustrating module-clinical trait relationships and gene significance were generated to identify key modules, with parameters set at GS > 0.5 and MM > 0.8 to ultimately determine the hub genes.

#### 2.1.4 Determination of hub genes

We further employed the JSVM-RFE algorithm for feature gene selection. The results intersected with the genes from the key modules identified in WGCNA and the DEGs, ultimately yielding the hub genes.

#### 2.1.5 Validation of hub genes

Firstly, we constructed a receiver operating characteristic (ROC) curve to validate the hub genes. Gene expression was then compared between the two groups using box plots for both experimental and validation datasets. Additionally, LASSO regression was utilized for cross-validation. We also utilized the GEPIA database (http://gepia.cancer-pku.cn/) to perform survival analysis, evaluating the diagnostic accuracy.

#### 2.1.6 GSEA analysis

GSEA was performed using gene sets that synergistically interact with the hub genes, allowing for the identification of enriched pathways associated with these gene sets.

#### 2.1.7 Immune infiltration analysis

Immune-related single-sample GESA (ssGSEA) enrichment analysis and CIBERSORT immune infiltration analysis were conducted. These two methods provided complementary insights into the immune landscape.

### 2.2 MR analysis

#### 2.2.1 Data sources

The IEU database (https://gwas.mrcieu.ac.uk/) served as the source for this portion of the data. The IL-2 dataset (GWAS ID: prot-c-3070_1_2) include 501,428 SNPs from a European population. The EBI GWAS Catalog (https://www.ebi.ac.uk/gwas/) were source of the data on AML and immune cells, with the AML accession number GCST90435652. Immune cell data were collected under accession numbers GCST90274758 to GCST90274848, encompassing 728 immune cell types along with their corresponding GWAS IDs (**Supplementary Table S1**), all derived from a European population.

#### 2.2.2 IVs selection and data harmonization

Genome-wide significant SNPs with a threshold of P < 5×10−8 were included. In the absence of such SNPs, we considered those with P < 5×10−6 as potential instruments. We clustered SNPs based on linkage disequilibrium (window size = 10,000 kb and r² < 0.001), excluding weak instrumental variables (F-statistics < 10).

#### 2.2.3 Primary analysis

We used inverse variance weighting (IVW) and MR-Egger methods as the primary methods for assessing causal relationships. Both methods needed to achieve a significance threshold of P < 0.05, and if neither method achieved this level, the IVW results were prioritized. IVW combines the causal effects represented by the Wald ratio of each SNP through meta-analysis, relying on the assumption that all SNPs are valid instruments. Therefore, this approach could be applied only after excluding SNPs exhibiting pleiotropy.

Firstly, the causal relationship between IL-2 and AML was evaluated using a two-sample bidirectional MR. Directionality was assessed using P values for either IVW or MR-Egger. If this condition was not met, the direction of overall effect was derived from the cumulative steps in the decomposition process, ensuring P values remained below 0.05 in each step, while also calculating the total utility.

#### 2.2.4 Mediation analysis

Bulk MR analyses were performed using 728 immune cell types as exposure and AML as the outcome, identifying immune cells that yielded significant results. IL-2 was treated as the exposure, with the selected immune cells as outcomes, identifying the double-positive immune cells. Refer to the method in the literature (21). We conducted a three-step MR analysis for mediation assessment. In the first step, IL-2 was used as the exposure, with the AML as the outcome to calculate the effect (beta_all). In the second step, IL-2 was used as the exposure, with the identified double-positive immune cells as the outcome to calculate the effect (beta1). In the third step, these double-positive immune cells served as the exposure, with AML as the outcome to calculate the effect (beta2). Different SNPs were utilized in each step to investigate whether immune cells mediate the association between IL-2 and AML. The overall effect of IL-2 on AML included its direct effect on AML and an indirect effect mediated through immune cells. The mediation effect was assessed as the indirect effect divided by the overall effect. Additionally, the delta method was employed to calculate the 95% confidence intervals (CI).

#### 2.2.5 Sensitivity analysis

We assessed heterogeneity and horizontal pleiotropy by calculating P values. P > 0.05 indicated no significant heterogeneity or pleiotropy. Outliers were removed when detected, and causal estimates were recalculated. If significant heterogeneity persists following removal, a random effects model would be applied to assess result stability, as this model is less sensitive to weak SNP-exposure associations. We also conducted a leave-one-out analysis to evaluate the impact of each SNP on the overall causal estimate.

### 2.3 Experimental validation

#### 2.3.1 Cell culture

This study protocol was reviewed and approved by Ethics Committee of Gaomi Maternity and Child Health Hospital, approval number 20230206-09. For studies in which human tissues, fluid, or cell lines were used, written informed consent was obtained from the donors’ parents to participate in the study. Donors’ parents signed an informed consent according to the principles outlined in the Declaration of Helsinki.

The human myeloid leukemia cells (KG-1a) were obtained from CELLCOOK (Guangzhou, Guangdong, China) and validated via STR analysis (**Supplementary Figure S1**). The control group consisted of human umbilical cord blood stem cells sourced from children at our institution who had consent from their legal guardians. A portion of these stem cells was sent to the Qilu Stem Cell Bank for testing, while the remainder was stored in liquid nitrogen in our laboratory. Successful testing by the Qilu Stem Cell Bank indirectly confirms the usability of the stem cells stored in our laboratory. The culture, cryopreservation, and passaging of KG-1a cells were conducted according to the product manual (**Supplementary Figure S2**). The cells (1 × 105) were cultivated in each well of six-well plates. In the experiment, cells (mRECs) from passages 3 to 6 are used.

#### 2.3.2 Plasmid construction

The Homo sapiens *CFD* gene sequence was retrieved and downloaded from NCBI (**Supplementary Table S2**). Primers were designed using the coding sequence (CDS) of the target gene, excluding the stop codon, and using XbaI and Eco53KI restriction sites, at both ends. Perform double enzyme digestion using XbaI restriction endonuclease (Biosharp, Shanghai, China) and Eco53KI restriction endonuclease (KALANG, Shanghai, China). Plasmids were constructed by restriction‐enzyme double digestion and ligation. Plasmid pBI121(HonorGene, Changsha, Hunan, China) was selected as the expression vector (**Supplementary Figure S3**).

The digested fragments and vectors were ligated to construct recombinant plasmids (**Supplementary Figure S4**). The ligation product was then transformed into the competent Escherichia coli DH5α cell (Whenzhou KeMiao Biological Technology Co.,Ltd, Wenzhou, Zhejiang, China), and the competent bacterial strain was revived on blasticidin-free media. A portion of the cells was plated on plates with kanamycin (Eta Biology,Beijing,China) resistance. After single colonies emerged, several were randomly selected for qPCR analysis following plasmid transfection into target cells. This process allowed for the assessment of hub gene expression in the target cells and confirmed the successful construction of the plasmids.

#### 2.3.3 Cell grouping and plasmid transfection

A total of three groups of cells were analyzed, including the normal group (human umbilical cord blood stem cells), the control group (KG-1a cells), and the experimental group (human umbilical cord blood stem cells transfected with plasmids). Each group had three compound holes. All groups were supplemented with 4500 µL of 20% DMEM culture medium (absin,Shanghai,China), 500 µL of fetal bovine serum(opcel,Shanghai,China), and 200 µL of P/S (penicillin and streptomycin) dual antibiotics (absin,Shanghai,China). Cells in the logarithmic growth phase were selected and transfected with plasmid DNA using Lipofectamine 2000 (Invitrogen,Hangzhou,Zhejiang,China),with three compound holes for each group.

#### 2.3.4 Real-time quantitative polymerase chain reaction

We extracted the total RNA from each cell group, and reverse transcription was performed to synthesize cDNA using specific primers (**Supplementary Table S3**). Data were analyzed using the 2-ΔΔCt method for quantification. GAPDH was the internal reference gene. The detection was performed with the Gentier 96E fluorescence quantitative PCR instrument made by TIANLONG, a company in China.

#### 2.3.5 Validation of hub gene

We used the RT-qPCR to compare the hub gene expression between the normal and control groups. After culturing cells for 72h, we utilized RT-qPCR to assess the hub gene expression in both the experimental group and the normal group. Subsequently, we replaced the fetal bovine serum in the culture medium with human serum, which was derived from residual blood collected post-transfusion in neonates with coagulation disorders at our institution. After an additional 72h, we performed RT-qPCR to evaluate the hub gene expression and the associated JAK-STAT and PI3K-Akt signaling pathways across all groups following induced overexpression. The experiment was repeated twice. Moreover, perform cell proliferation assays using the MTT Cell Proliferation and Cytotoxicity Detection Kit - 500T (Wanlei Biotechnology, Shanghai, China) and the Multiskan™ FC microplate reader (ThermoFisher, USA). Three parallel holes were set in each group, and the experiment was repeated three times. Referring to the literature (22), the concentration of MTT is 0.1 mg/mL; the wavelength of transmitted light is 565 nm. In 96-well plates, 5000 cells are seeded in each well.

### 2.4 Statistical analysis

Statistical analyses were performed using SPSS 18.0 and R 4.1.1. P<0.05 was considered statistically significant. Comparisons between two groups were conducted using an independent samples t-test, while pairwise comparisons among multiple groups were conducted using the LSD-t test.

## 3 Results

### 3.1 Transcriptomic features

**Figure 1:**
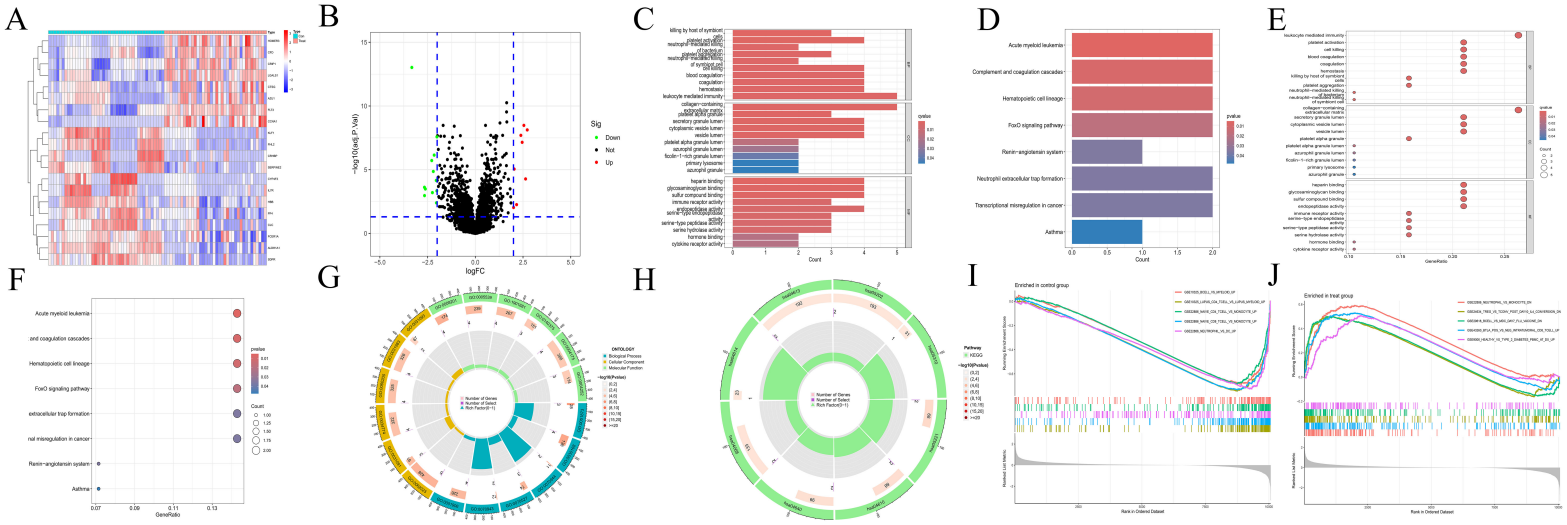
Transcriptomics analyses. **(A)** The heatmap illustrating DEGs in CH and normal samples. The abscissa indicates different samples. Blue represents the normal group (con) and red represents the CH group (Treat). The ordinate represents genes. High expression is indicated in red and low expression is shown in deep blue. **(B)** The volcano plot displaying DEGs between CH samples and normal samples. Red points, green points, and black points indicate genes that are up-regulated, down-regulated, or have no significant difference in CH compared with the normal group. **(C, D)** Bar plots for GO and KEGG enrichment analyses. The redder the color, the more significant the difference, and the bluer the color, the lower the difference. The length of the bars represents the number of enriched genes. **(E, F)** Bubble plots for GO and KEGG enrichment analyses. The redder the color, the more significant the difference, and the bluer the color, the lower the difference. The size of the bubbles represents the number of enriched genes. **(G, H)** Circle plots for GO and KEGG enrichment analyses. The outermost circle represents the GO IDs, and the next inner circle represents the number of enriched genes. The following inner circle represents the number of differentially expressed genes, and the innermost circle represents gene proportions. The color represents the second circle from outside to inside. The redder the color, the more significant the differential gene enrichment is. **(I, J)** The active gene sets in normal and CH samples in GSEA analysis. The abscissa denotes gene ranking, while the ordinate represents enrichment scores. Only the top five gene sets with the most significant enrichment are presented.

A total of 20 differentially expressed genes (DEGs) were identified between the acute myeloid leukemia group (denoted as “treat”) and the normal group (denoted as “con”). Specifically, 8 genes were found to be upregulated (with log2 fold change (log2FC) > 2), namely CTSG,CRIP1,AZU1,HOMER3,LGALS1,FLT3,CFD, and CCNA1. Meanwhile, 12 genes were downregulated (log2FC < -2) (as shown in **Figures 1A, B**), which included ALDH1A1, CLC, HBB, CRHBP, KLF1, CYP4F3, SERPINE2, FHL2, PF4, IL7R, FCER1A, and SDPR. The Gene Ontology (GO) enrichment analysis (depicted in **Figures 1C, E, G**) revealed that these DEGs were associated with processes such as “killing by host of symbiont”, “neutrophil - mediated killing”, “leukocyte mediated immunity”, “immune receptor activity”, “platelet activation”, “blood coagulation”, and “hemostasis”. This strongly suggests a close and intricate relationship between these genes and the immune system as well as blood coagulation. The Kyoto Encyclopedia of Genes and Genomes (KEGG) enrichment analysis (illustrated in **Figures 1D, F, H**) demonstrated that the DEGs were related to pathways like “Acute myeloid leukemia”, “Complement and coagulation cascades”, and “Hematopoietic cell lineage”. Evidently, this indicates a significant connection with the blood system. Furthermore, the immune-related Gene Set Enrichment Analysis (GSEA) uncovered some interesting findings. In the control group (**Figure 1I**), there was an upregulation associated with gene sets such as BCELL, MYELOID, MYELOID, LUPUS, MONOCYTE, CD4_TCELL, CD8_TCELL, and NEUTROPHIL. In contrast, in the AML group (**Figure 1J**), there was a downregulation related to gene sets including NEUTROPHIL, MONOCYTE, TREG, TCONV, IL4, BCELL, MDC, etc., along with an upregulation related to gene sets like BTLA and CD8_TCELL.

### 3.2 Screening of hub gene

**Figure 2:**
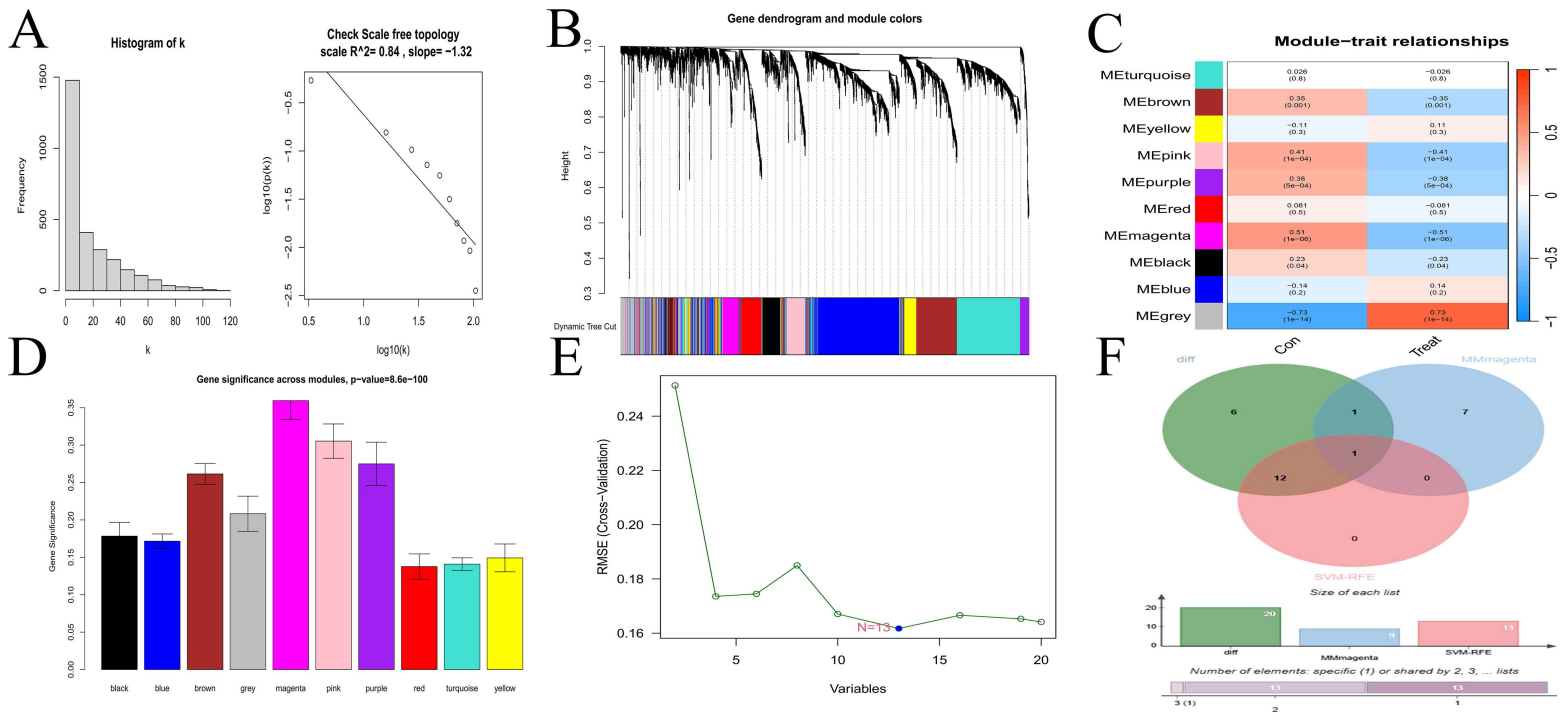
Selection of hub gene. **(A)** Scale-free network topology. **(B)** Merged weighted gene co-expression network. **(C)** Heatmap illustrating the correlation between modules and clinical traits. Red represents a positive correlation, while blue represents a negative correlation. For the values within the grids, the number above stands for the correlation coefficient. A positive number indicates a positive correlation, and a negative number indicates a negative correlation. The number below represents the P-value, and the smaller the P-value is, the higher the correlation. **(D)** Gene importance plot. The abscissa represents module names, and the ordinate represents gene importance. The higher the value is, the more important the gene is. **(E)** SVM-RFE for predicting hub genes. The abscissa shows the variation in the number of genes, and the ordinate shows the cross-validation error. **(F)** Venn diagram depicting the hub genes from three gene sets.

The scale-free topological network revealed a correlation coefficient of 0.84 (**Figure 2A**), exceeding the threshold of 0.8, thereby confirming that the selected power value effectively constructed a scale-free network. The results from WGCNA module analysis are presented in **Figure 2B**. The correlation analysis between modules and clinical traits (**Figure 2C**) revealed that the grey and magenta modules had the smallest P-values, indicating the highest correlations. The gene importance analysis (**Figure 2D**) demonstrated that the magenta module was the most significant one. After comprehensive evaluation, the magenta module was finally determined to be the most relevant and important module. The SVM-RFE method located the point with the minimum cross-validation and marked it. This encompassed 13 characteristic genes (**Figure 2E**), suggesting that these 13 genes had the highest importance for the classification task. Finally, the common gene identified by taking the intersection of the 20 DEGs, the gene set of the magenta module, and the 13 characteristic genes was CFD (**Figure 2F**). WGCNA is a method for analyzing gene expression patterns in multiple samples. It can cluster genes based on similar gene expression patterns and form modules, and then analyze the relationships between modules and specific traits (such as patients’ clinical information) (23). Through this method, we found the module and its gene set that were most relevant to the disease. However, the gene set obtained by this method could not effectively distinguish different disease types and lacked gene specificity for disease diagnosis. On the other hand, the Support Vector Machine - Recursive Feature Elimination (SVM-RFE) analysis is a supervised machine learning technique used to identify the optimal core genes by removing the feature vectors generated by SVM (24). Through this method, we found the gene set that was most important for disease classification and typing, which had high sensitivity for classification and typing but lacked connections with biological processes. Finally, we took the intersection of the gene sets obtained by multiple methods, which compensated for their respective shortcomings and led to higher accuracy of the obtained hub genes.

### 3.3 Validation of hub gene and GSEA enrichment analysis of synergistic genes

**Figure 3:**
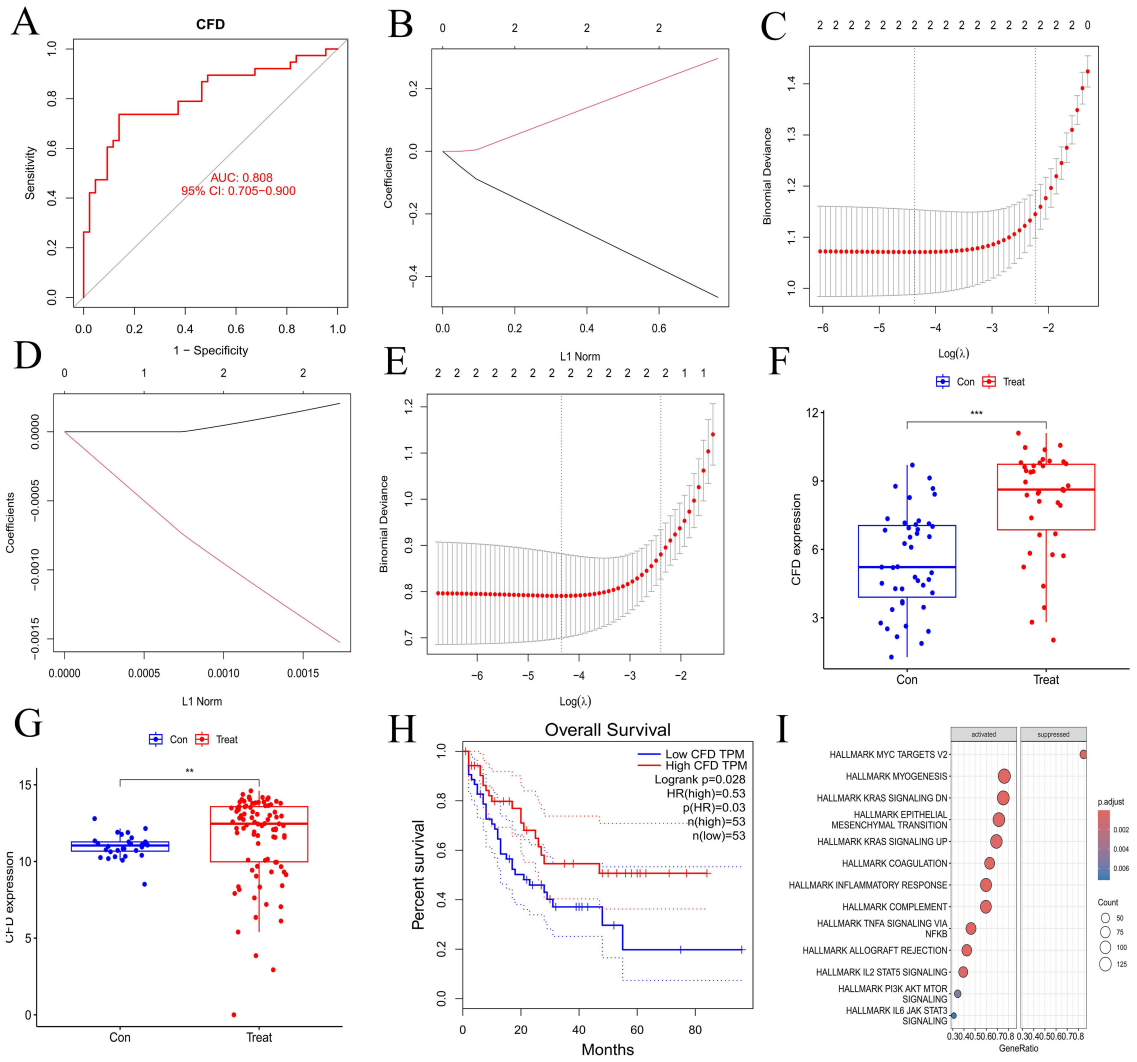
Validation of hub gene and GSEA enrichment analyses of co-expressed genes. **(A)** ROC curve for hub gene. The abscissa represents the false positive rate (1 - specificity) and the ordinate represents the true positive rate (sensitivity). **(B, D)** Lasso regression analyses for the experimental and validation datasets. **(C, E)** Cross-validation plots for lasso regression in experimental and validation datasets. The abscissa indicates Log(λ) values, and the ordinate indicates cross-validation errors. **(F, G)** Box plots illustrating the differential expression of hub gene in experimental and validation datasets. The abscissa represents the group classification, and the ordinate indicates the expression levels of hub gene. **(H)** Survival analysis for hub gene. **(I)** Immune-related GSEA enrichment analysis of co-expressed hub gene. "***", "**", "*" correspond to 0.001, 0.01 and 0.05 respectively.

We validated the gene from multiple perspectives. First, the ROC curve of *CFD* gene was drawn (**Figure 3A**), and the area under the curve (AUC) was greater than 0.8. Subsequently, we applied Lasso regression in both the experimental and validation datasets (**Figures 3B–E**), which identified a minimal subset of two genes through cross-validation, including *CFD*. In terms of *CFD* gene expression, the two sample groups showed a significant difference based on boxplot analyses (**Figures 3F, G**). Survival analysis indicated that *CFD* gene expression significantly influenced survival outcomes in AML compared to normal samples (**Figure 3H**). This had indicated that altering the gene expression status of CFD was beneficial for improving the clinical prognosis of patients.

Through GSEA analysis, we identified pathways that are enriched with synergistic genes associated with the *CFD* gene (**Figure 3I**), including INFLAMMATORY RESPONSE, TNFA SIGNALING VIA NFKB, IL2 STAT5 SIGNALING, PI3K AKT MTOR SIGNALING, and IL6 JAK STAT3 SIGNALING. The results demonstrated that the high expression of CFD could promote the inflammation response mediated by IL-2 and IL-6, as well as the activation of PI3K/AKT and JAK/STAT3 signaling transduction pathways. This suggests that CFD might affect the clinical prognosis of AML by promoting these pathways such as IL-2, PI3K/AKT, and JAK/STAT3.

### 3.4 Immune infiltration analysis

**Figure 4:**
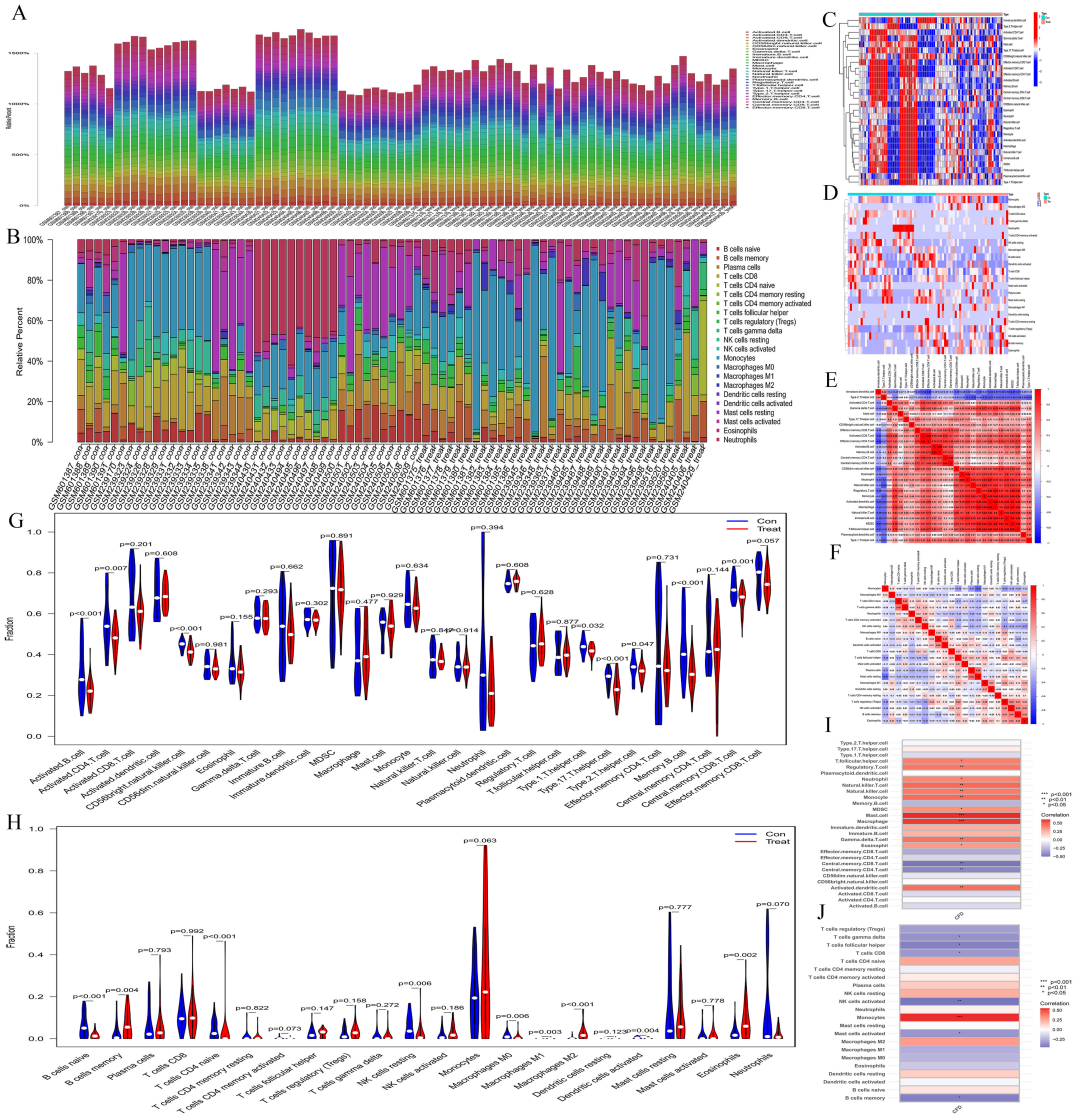
Immune infiltration analyses. **(A, B)** Bar plots depicting immune-related enrichment analyses via ssGSEA and immune infiltration assessment using CIBERSORT. The abscissa represents sample names, and the ordinate represents the percentage of immune cells. **(C, D)** Heatmaps generated from ssGSEA and CIBERSORT analyses. The abscissa represents different samples. Blue represents the normal group (con) and red represents the CH group (Treat). The ordinate represents various immune cell types. High expression is indicated in red and low expression is shown in deep blue. **(E, F)** Heatmaps illustrating the correlation between immune cells based on ssGSEA and CIBERSORT. The red indicates a higher positive correlation, and the deeper blue reflects a higher negative correlation between the two variables. **(G, H)** Violin plots of immune cell distributions for both ssGSEA and CIBERSORT analyses. The abscissa represents immune cell types, while the ordinate indicates the percentage of immune cells. Blue represents the control group, and red represents the CH group, and P-values indicate the statistical significance of differences between the two groups. **(I, J)** Heatmaps of immune cell correlation analysis. The abscissa represents gene names, while the ordinate indicates immune cell types. The red indicates a higher positive correlation, and the deeper blue reflects a higher negative correlation between the two variables.

The bar charts revealed that the immune cell compositions among samples are largely consistent (**Figures 4A, B**). However, AML and normal groups exhibited significant differences in both quantity and composition of immune cells (**Figures 4C, D**). The correlation heatmaps provide insights into the association among different immune cell types (**Figures 4E, F**). The violin plots illustrate notable differences in proportions of various immune cell types (**Figures 4G, H**), including activated B cells, activated CD4 T cells, CD56 bright natural killer cells, type 1 T helper cells, type 17 T helper cells, type 2 T helper cells, memory B cells, central memory CD8 T cells, naive B cells, memory B cells, naive CD4 T cells, resting NK cells, M0 macrophages, M1 macrophages, M2 macrophages, activated Dendritic cells, and eosinophils between the normal and AML groups. Furthermore, the immune cell correlation analyses indicated that, according to the ssGSEA method, *CFD* gene was highly positively correlated with mast cells and macrophages, while exhibiting a strong negative correlation with central memory CD8 T cells and central memory CD4 T cells. In the CIBERSORT method, *CFD* gene showed a high positive correlation with monocytes and a strong negative correlation with activated NK cells (**Figures 4I, J**). These analyses effectively evaluated the tumor microenvironment of AML and indicated a significant immunological difference between AML and normal cells, suggesting a correlation between AML cells and immune cells. Meanwhile, our results also confirmed a strong association between CFD and immune cells.

### 3.5 MR analysis

A Bulk MR analysis of 728 immune cell types identified 27 positive immune cells (**Supplementary Table S4**). IL-2 has been determined to have a role in AML. The Bulk MR analysis of immune cells identified CD27 on CD24+ CD27+ B cell (GWAS ID: ebi-a-GCST900017983) as the dual-positive immune cell. Our results demonstrated that 27 types of immune cells had an impact on the occurrence and development of AML, but only CD27 on CD24+ CD27+ B cell was regulated by IL-2.

**Figure 5:**
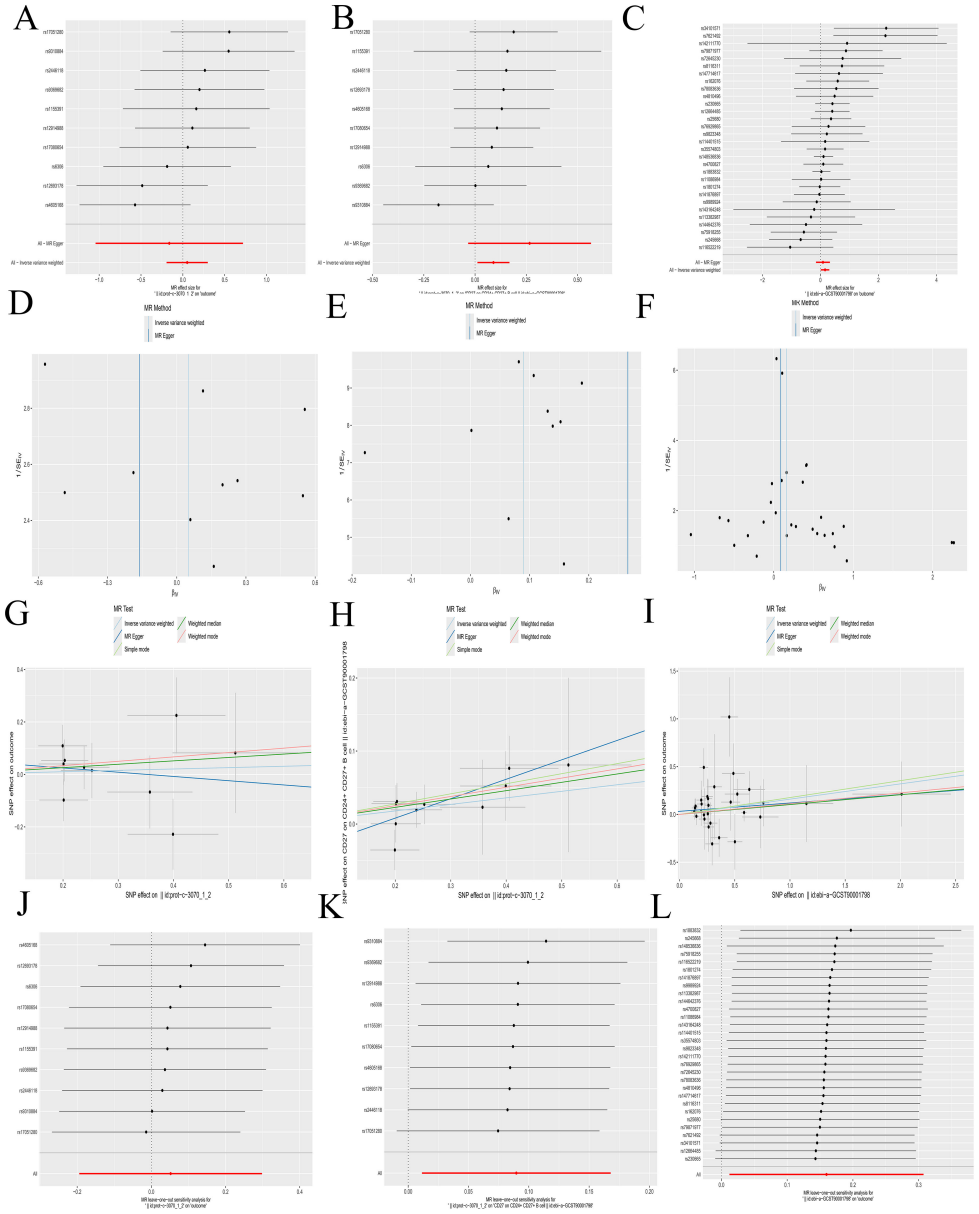
Mendelian randomization analyses. **(A-C)** Forest plots illustrating the results of each analytical step. **(D-F)** Funnel plots corresponding to each step of the analysis. **(G-I)** Scatter plots displaying the relationships between variables. **(J-L)** Leave-one-out forest plots summarizing the robustness of the findings. The following groups of exposure and outcomes: (1) Exposure to IL-2, outcome AML: **(A, D G, J)**. (2) Exposure to IL-2, outcome CD27 on CD24+ CD27+ B cell: **(B, E, H, K)**. (3) Exposure to CD27 on CD24+ CD27+ B cell, outcome: AML: **(C, F, I, L)**.

The role of IL-2 in AML is a promoting effect (**Figures 5A, G**). The overall effect estimate (beta_all) is 0.052, indicating no significant heterogeneity (Mendelian randomization Egger test method, P = 0.317; inverse variance weighted method, P = 0.385) or pleiotropy (P = 0.632).IL-2 exhibited a positive correlation with the dual positive immune cell (**Figures 5B, H**), with a beta estimate (beta1) of 0.090 (IVW method, OR=1.094; [95% CI, 1.011-1.183], P=0.025), and no significant heterogeneity (MR Egger method, P=0.827; IVW method, P=0.764) or pleiotropy (P=0.267). The dual positive immune cell was also positively associated with AML (**Figures 5C, I**), yielding a beta estimate (beta2) of 0.160 (IVW method, OR=1.173; [95% CI, 1.012-1.360], P=0.034), and no significant heterogeneity (MR Egger method, P=0.665; IVW method, P=0.685) or pleiotropy (P=0.462) was observed.

Further calculations revealed a mediating effect of 0.0144 (beta12 = beta1 x beta2) and a direct effect of 0.0376 (beta_dir = beta_all - beta12), with the mediating effect contributing to 27.69% of the total effect (beta12_p = beta12/beta_all). The funnel plots displayed a symmetric distribution without apparent outliers, suggesting minimal heterogeneity (**Figures 5D–F**). The leave-one-out forest plots indicated the robustness of the results, with no significant outliers detected (**Figures 5J–L**). Scatter plots and corresponding odds ratios (OR) suggest that IL-2 influences AML by acting on CD27 on CD24+ CD27+ B cells. Previously, we have confirmed that CFD has a promoting effect on IL-2. The results here further verified that IL-2 promotes the occurrence and development of AML by acting on CD27 on CD24+ CD27+ B cell. This forms a complete immunoregulatory pathway through which CFD promotes the occurrence and development of AML, which is of great value for guiding clinical immunotherapy in the future.

Details regarding all exposure data SNPs involved in the above steps can be found in **Supplementary Table S5**. Results of the five MR methods were presented in **Supplementary Table S6**, and results of individual SNP analyses were detailed in **Supplementary Table S7**.

### 3.6 Experimental validation

**Figure 6:**
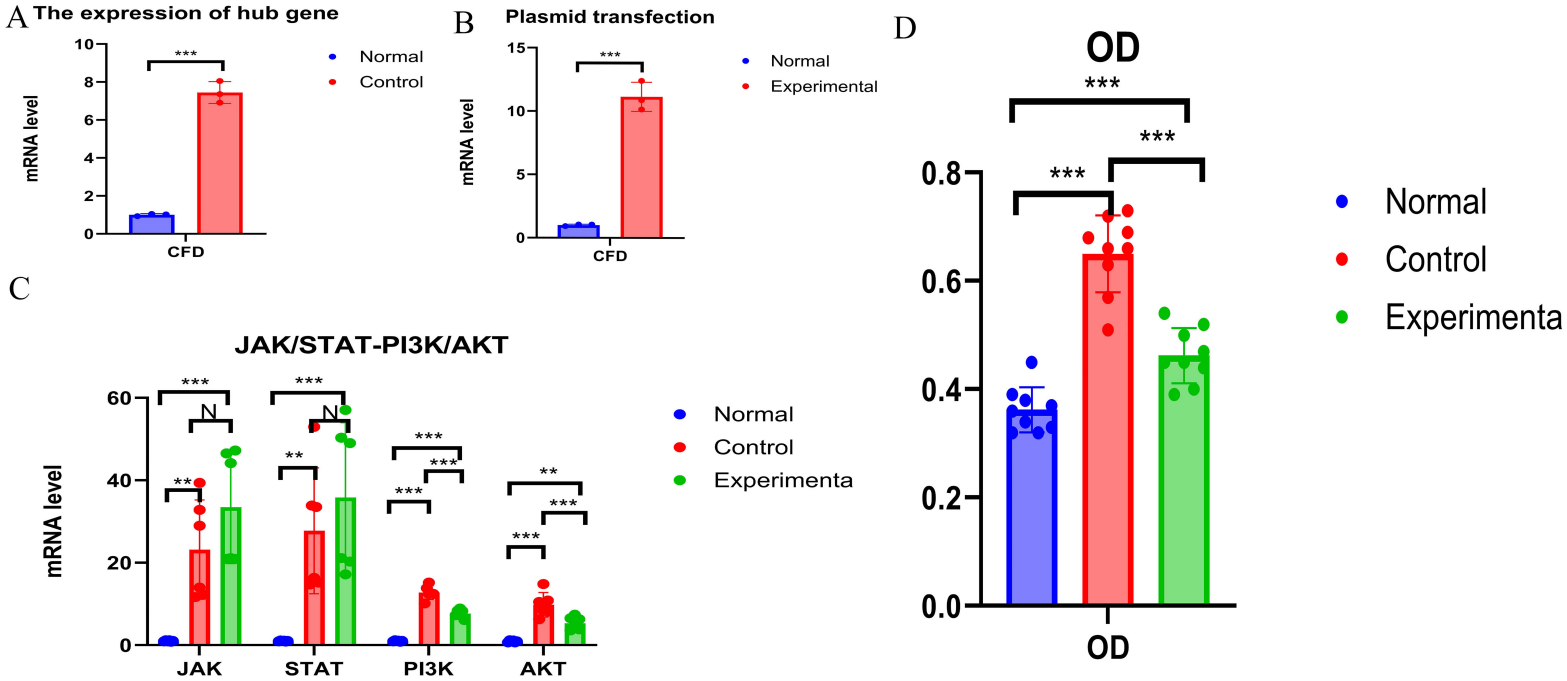
Experimental Validation **(A)** The expression of CFD. **(B)** The expression status of CFD after transfection. **(C)** Changes of JAK/STAT and PI3K - AKT Before and After Transfection. **(D)** Comparison of OD Values among Different Groups. "***", "**", "*" correspond to 0.001, 0.01 and 0.05 respectively.

Consistent with the bioinformatics results, the expression of *CFD* gene in AML had been confirmed to be significantly elevated compared to the normal group (**Table 1**, **Figure 6A**). Successful plasmid transfection led to an upregulation of *CFD* gene expression in normal cells (**Table 2**, **Figure 6B**). In AML cells, both the PI3K/Akt and JAK/STAT signaling pathways were relatively highly expressed, showing a strong correlation with *CFD*. Following the induction of high *CFD* gene expression, JAK/STAT and PI3K/Akt pathways were simultaneously activated, with JAK/STAT exhibiting a more pronounced increase (**Table 3**, **Figure 6C**). The MTT assay for assessing cell proliferation revealed statistically significant differences among the groups (**Table 4**, **Figure 6D**), suggesting a potential role for *CFD* gene in regulating cell proliferation. Although the induction of *CFD* gene in normal cells enhanced their proliferative capacity, the extent of this enhancement was limited and did not reach the level observed in AML cells.

**Table 1:** The expression of hub gene.

	Normal Group	Control group	t	*P*
CFD	1.001 ± 0.061	7.444 ± 0.574	-19.333	0.000

**Table 2:** Plasmid transfection.

	Normal Group	Experimental group	t	*P*
CFD	1.002 ± 0.069	11.146 ± 1.153	-15.211	0.000

**Table 3:** Changes in JAK-STAT and PI3K-Akt pathways after high expression of CFD.

	Group	Mean 1	Mean 2	S1	S2	*P*
JAK	Normal vs. Control	1.010	23.172	0.153	12.036	0.002
	Normal vs. Experimenta	1.010	33.484	0.153	13.725	0.000
	Control vs. Experimenta	23.172	33.484	12.036	13.725	0.111
STAT	Normal vs. Control	1.003	27.767	0.085	15.271	0.004
	Normal vs. Experimenta	1.003	35.838	0.085	18.115	0.001
	Control vs. Experimenta	27.767	35.838	15.271	18.115	0.323
PI3K	Normal vs. Control	1.005	12.754	0.115	1.678	0.000
	Normal vs. Experimenta	1.005	7.691	0.115	1.007	0.000
	Control vs. Experimenta	12.754	7.691	1.678	1.007	0.000
Akt	Normal vs. Control	0.904	9.807	0.207	2.975	0.000
	Normal vs. Experimenta	0.904	5.307	0.207	1.592	0.001
	Control vs. Experimenta	9.807	5.307	2.975	1.592	0.001

**Table 4:** Cell OD statistics.

Group (OD)	Mean 1	Mean 2	S1	S2	*P*
Normal vs. Control	0.362	0.650	0.042	0.071	0.000
Normal vs. Experimenta	0.362	0.462	0.042	0.051	0.001
Control vs. Experimenta	0.650	0.462	0.071	0.051	0.000

## 4 Discussion

The identification of genetic abnormalities plays a crucial role in the diagnosis, prognosis and classification of AML (25, 26). The inflammatory microenvironment has long been regarded as promoting tumorigenesis in solid cancers (27, 28). However, it was not until recently that the important role of inflammation and immunity in hematological malignancies was discovered (26, 29). First, we screened out the hub gene as *CFD* gene. Through enrichment analysis, it was determined that it has the effect of promoting IL-2. With IL-2 as an intermediary, on the one hand, IL-2 can promote the occurrence and development of AML by promoting CD27 on CD24+CD27+ B cells. On the other hand, IL-2 can also promote the proliferation of AML cells by activating the JAK/STAT and PI3K/Akt pathways.

The *CFD* gene we screened out in our research is a crucial regulator of immune response, encoding a member of the serine peptidase S1 family or the chymotrypsin-like protease family. These proteins catalyze the cleavage of factor B, serving as a rate-limiting step in the alternative pathway of complement activation (30). Studies have shown that *CFD* can serve as a reliable prognostic marker for AML (31). However, research on the mechanism by which *CFD* gene acts on AML is insufficient.

We found that the *CFD* gene is primarily linked to inflammation pathways associated with IL-2 and IL-6, as well as to the PI3K/AKT and JAK/STAT3 signaling pathways. CFD represents the bottleneck in convertase formation (32), and convertase is the most important enzyme in regulating the alternative pathway of complement activation (33), which will ultimately lead to the production of molecules such as C3b, C3a, and C5a (34). C5a can induce mast cells (35) and neutrophils (36) to secrete IL-6 and promote T lymphocytes (37) to produce IL-2, which is consistent with the results of our study.

Immune infiltration analysis demonstrated a strong positive correlation between *CFD* gene and mast cells, macrophages, and monocytes, while revealing a strong negative correlation with central memory CD8+ T cells, central memory CD4+ T cells, and activated NK cells. This has some consistent points with the results of previous studies which found that there is a close relationship between IL-6, T cells, NK cells and AML (38, 39). Based on our bioinformatics results, IL-6 and IL-2 are mediator factors worthy of in-depth study in this research. However, using the MR method, we only found a connection between IL-2 and AML. Moreover, IL-2 is often studied as an immunotherapeutic approach for treating AML (40, 41). Therefore, we will focus on IL-2 as the key of our follow-up research.

Our subsequent in-depth research shows that *CFD* gene ultimately promotes the progression of AML by activating IL-2 and then activating CD27 on CD24+ CD27+ B cells.IL-2 can regulate B cells, which is consistent with previous studies (42, 43). However, our research goes further. We have found the type of B cells most relevant to the development of AML. At the same time, *in vitro* experiments show that IL-2 can play a promoting role such as promoting AML proliferation through the JAK/STAT and PI3K/Akt pathways. Multiple studies have shown that IL-2 mainly activates three signaling pathways: JAK/STAT, ERK, and PI3K (44–47). This is highly consistent with our research results. It promotes the proliferation and activation of regulatory T cells (Tregs), enhancing their immunosuppressive function, which indirectly facilitates tumor cell growth by inhibiting effective anti-tumor immune responses (48). Furthermore, IL-2 may stimulate tumor cells to secrete certain angiogenic factors, promoting the formation of new blood vessels within tumors. Adequate oxygen and nutrients help tumor cells grow and spread (49). Some tumor cells express IL-2 receptors, and upon binding with IL-2, intracellular signaling pathways are activated, such as the PI3K/Akt pathway, which promotes tumor cell survival and proliferation. All of these are theoretically consistent with our research result that IL-2 has a promoting effect on AML. At the same time, combined with these studies, we can reasonably infer that *CFD* gene realizes the subsequent activation of the JAK/STAT and PI3K/Akt pathways by first activating IL-2.

The JAK/STAT pathway is involved in various physiological processes, including cell proliferation, differentiation, apoptosis, immune regulation, and inflammatory responses. For instance, it is crucial to the development and functional regulation of immune cells, as well as in cellular responses to growth factors (50–53). The PI3K/Akt pathway primarily responds to extracellular signals, promoting metabolism, proliferation (54), cell survival (55), growth, and angiogenesis, and has also been implicated in cancer progression (56, 57). These characteristics of these two pathways are consistent with the role of promoting the proliferation of AML cells confirmed by our *in vitro* experiments.

Our study still has certain limitations. First, the results of the MR analysis have not been experimentally verified. Second, the MR data is from the European population and lacks data from other populations.

Our study has confirmed the impact of the three subsequent immune pathways dominated by *CFD* gene and mediated by IL-2 on AML. This is undoubtedly of great significance for enriching the immune theory related to AML and improving its related immunotherapy.

## 5 Conclusion

**Figure 7:**
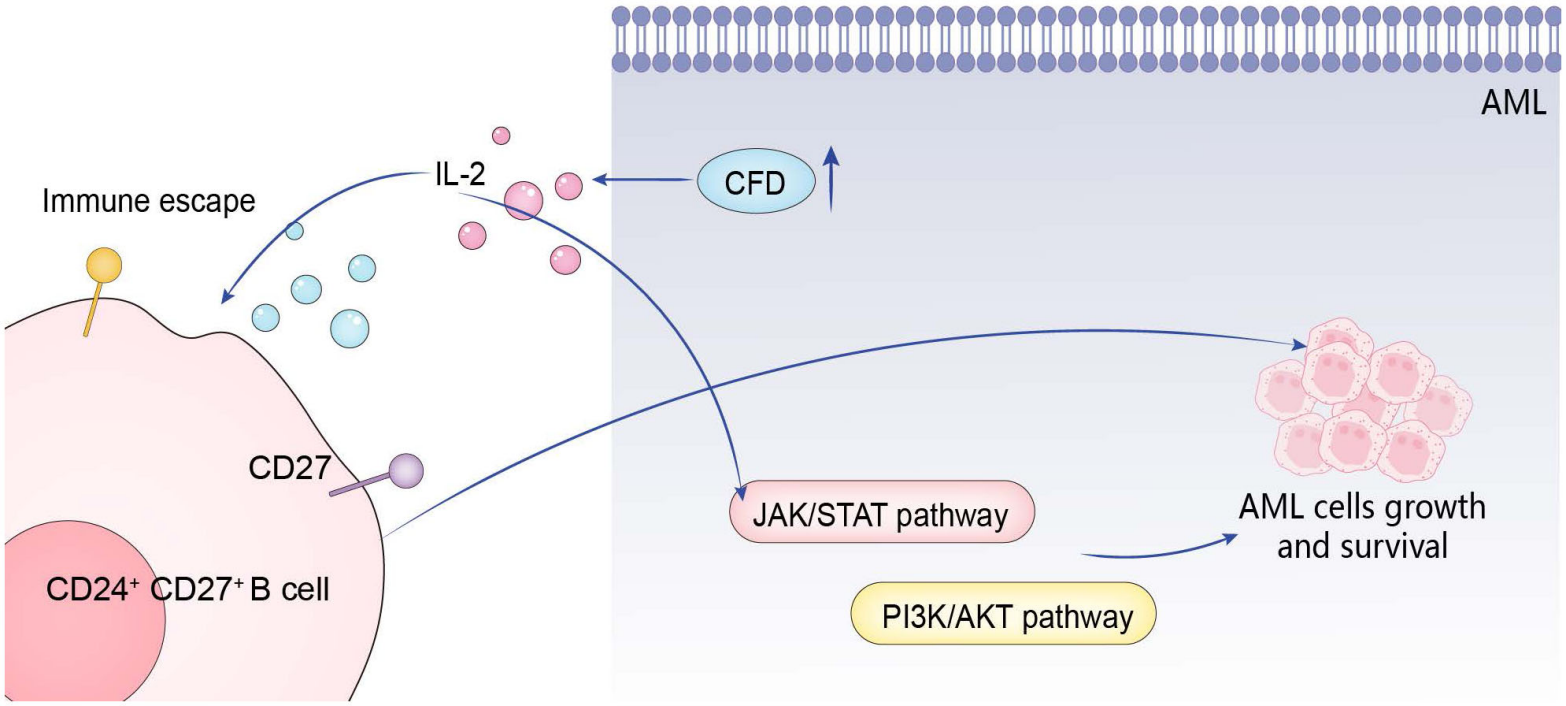
Action mechanism of hub gene.

The abnormal high expression of *CFD* gene first activates IL-2, and then promotes the occurrence and development of AML through the positive effects on three pathways: CD27 on CD24+ CD27+ B cell, JAK/STAT, and PI3K/Akt (**Figure 7**).

## Data Availability

Publicly available datasets were analyzed in this study. This data can be found here: https://www.ncbi.nlm.nih.gov/geo/query/acc.cgi?acc=GSE9476; https://www.ncbi.nlm.nih.gov/geo/query/acc.cgi?acc=GSE24395; https://www.ncbi.nlm.nih.gov/geo/query/acc.cgi?acc=GSE30029; https://gwas.mrcieu.ac.uk/datasets/prot-c-3070_1_2/ https://www.ebi.ac.uk/gwas/accession number: GCST90435652,GCST90274758 to GCST90274848.
