# Corrigendum: Identification of hub genes and immune-related pathways in acute myeloid leukemia: insights from bioinformatics and experimental validation

**DOI:** 10.3389/fimmu.2025.1629151

**Published:** 2025-05-28

**Authors:** Mingliang Shan, Li Xu, Wenzhe Yang, Shiguo Liu, Zhaoqing Cui

**Affiliations:** ^1^ Postdoctoral Workstation, Liaocheng People’s Hospital, Liaocheng, China; ^2^ Postdoctoral Mobile Stations, The Affiliated Hospital of Qingdao University, Qingdao, China; ^3^ Post - Doctoral Innovation Practice Base, Gaomi Maternity and Child Health Hospital, Gaomi, China; ^4^ School of Management, Shandong Second Medical University, Weifang, China; ^5^ College of Acupuncture and Massage, Shandong University of Traditional Chinese Medicine, Jinan, China

**Keywords:** AML, hub gene, bioinformatics, machine learning, Mendelian randomization, plasmid, RT-qPCR

In the published article, there was an error in Affiliations 3–5. Instead of 

“^3^School of Management, Shandong Second Medical University, Weifang, China

^4^College of Acupuncture and Massage, Shandong University of Traditional Chinese Medicine, Jinan, China

^5^Post - Doctoral Innovation Practice Base, Gaomi Maternity and Child Health Hospital, Gaomi, China”.

it should be 

“^3^Post - Doctoral Innovation Practice Base, Gaomi Maternity and Child Health Hospital, Gaomi, China

^4^School of Management, Shandong Second Medical University, Weifang, China

^5^College of Acupuncture and Massage, Shandong University of Traditional Chinese Medicine, Jinan, China”.

In the published article, there was an error in Correspondence, Instead of “lccuizhaoqing123@163.com”, it should be “cuizhaoqing203@163.com”

The authors apologize for this error and state that this does not change the scientific conclusions of the article in any way. The original article has been updated.

