# Supplementary material for: Identification of hub genes and immune-related pathways in acute myeloid leukemia: insights from bioinformatics and experimental validation
Source: Front Immunol. 2025 Jan 10;15:1511824. doi: 10.3389/fimmu.2024.1511824 (PMC11757261; doi:10.3389/fimmu.2024.1511824)

STR Identification of KG-1a Cells

Experimental Procedures

Use the Vazyme Cell Genomic DNA Extraction Kit to extract the sample DNA. Then, use the Microread Human STR Locus Detection Kit to amplify the sample. Conduct signal collection through the Applied Biosystems SeqStudio Genetic Analyzer, and perform locus analysis using Genemapper software 6.

Prepare the reaction system in PCR tubes according to the following system:

component	Volume (Content)
PCR Master Mix	5.0 μl
Primer Mix	2.5 μl
sample	30 ng
DEPC water	make up to 10.0 μl

Carry out the amplification reaction in a PCR instrument. The reaction program is as follows:

temperature	time	cycle
95℃	5 min	N/A
94℃	10 s	28-30 cycles
61℃	1 min	
70℃	30 s	
60℃	15 min	N/A

Prepare the electrophoresis samples in PCR tubes: denature at 95 °C for 3 minutes → cool at 4 °C for 3 minutes, and then load the samples onto the instrument to collect data.

**component**	**volume**
Hi-Di™ Formamide	8.5 μl
SIZE	0.5 μl
sample	1.0 μl

experimental results

Sample number	database	Matched Cells	matching degree	instruction
KG-1a	ATCC, DSMZ	KG-1a	100%	匹配

Analysis of the Reported
Values of Sample Loci

Loci	KG - 1a Sample STR Report Value			KG-1a Database Reference Values		
	AL1	AL2	AL3	AL1	AL2	AL3
**Amel**	X	Y		X	Y	
**D3S1358**	15	16		15	16	
**TH01**	7	8		7	8	
**D21S11**	28	29		28	29	
**D18S51**	10.2	18		10.2	18	
**Penta E**	7	13		7	13	
**D5S818**	13			13		
**D13S317**	11	12		11	12	
**D7S820**	8	10		8	10	
**D16S539**	10	11		10	11	
**CSF1PO**	7			7		
**Penta D**	8	9		8	9	
**vWA**	14	19		14	19	
**D8S1179**	13	14		13	14	
**TPOX**	7	9		7	9	
**FGA**	22			22		
**D19S433**	11	16.2		11	16.2	
**D12S391**	21					
**D6S1043**	18	19				
**D2S1338**	20	23		20	23	
**D1S1656**	14	16.3				

Cell STR Peak Chart

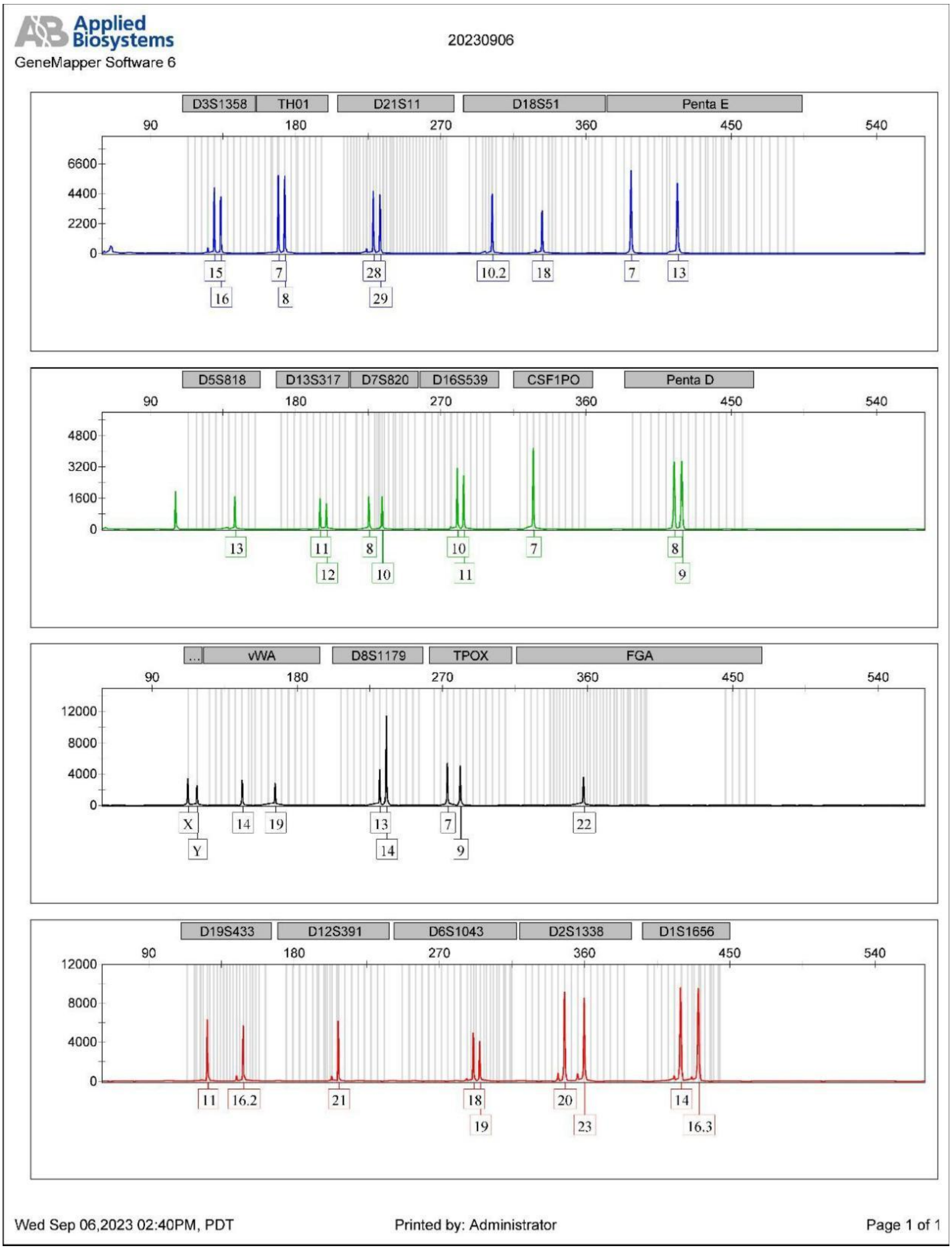

Supplement: Supplementary file 1 [file DataSheet1.pdf]
