# Supplementary figures and images for: Identification of hub genes and immune-related pathways in acute myeloid leukemia: insights from bioinformatics and experimental validation
Source: Front Immunol. 2025 Jan 10;15:1511824. doi: 10.3389/fimmu.2024.1511824 (PMC11757261; doi:10.3389/fimmu.2024.1511824)

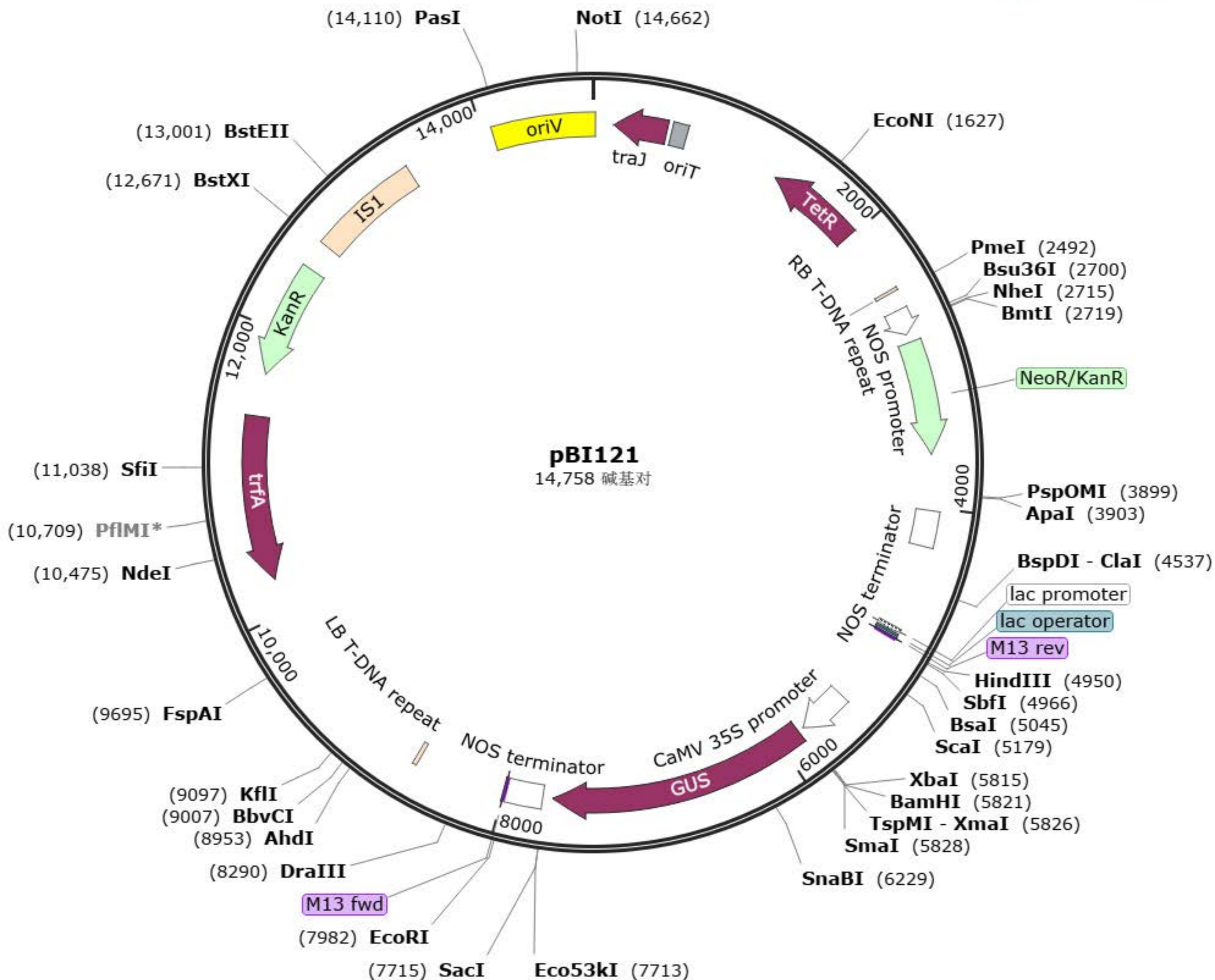

Supplement: Supplementary file 3 [file DataSheet3.pdf]

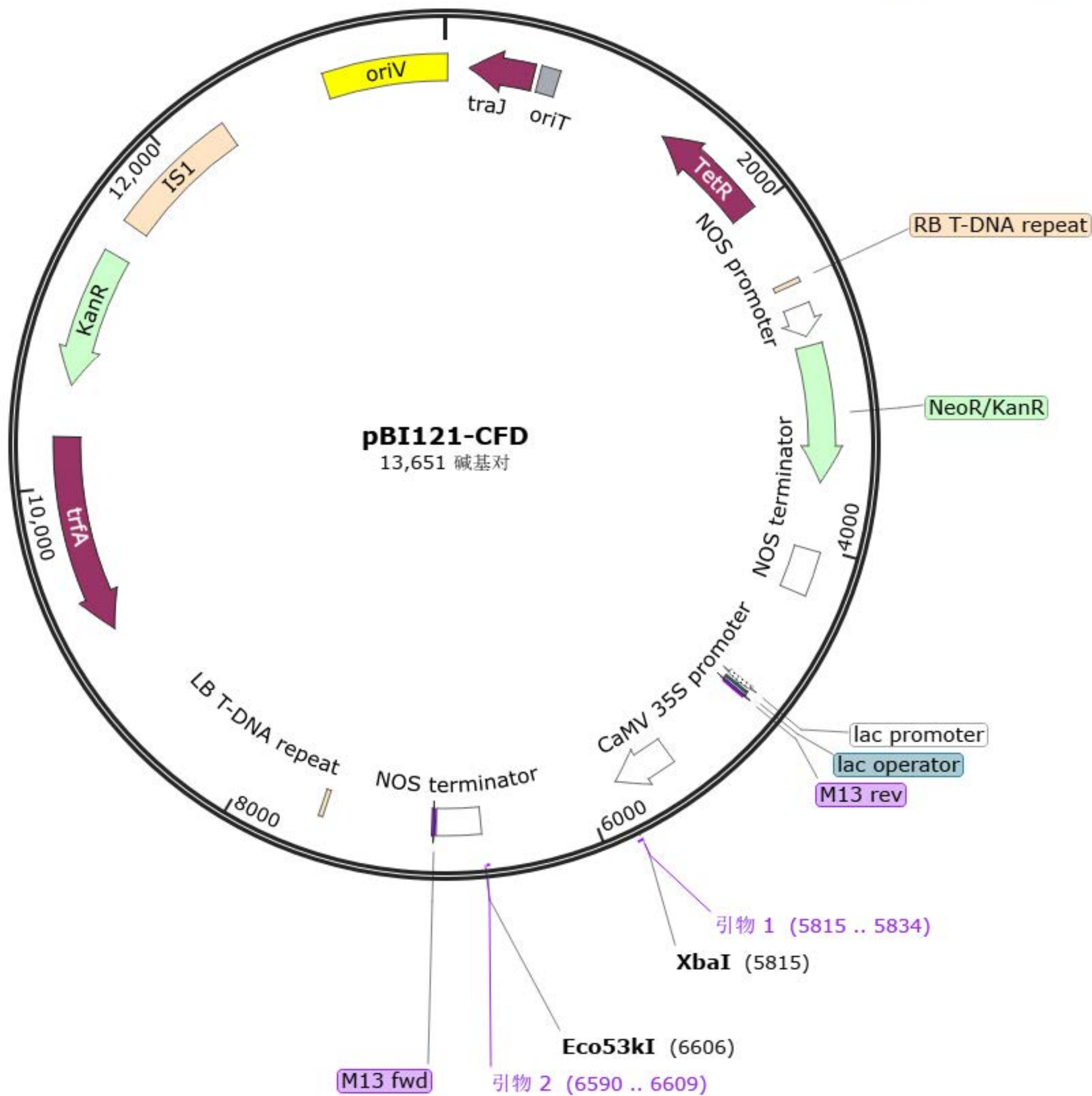

Supplement: Supplementary file 4 [file DataSheet4.pdf]

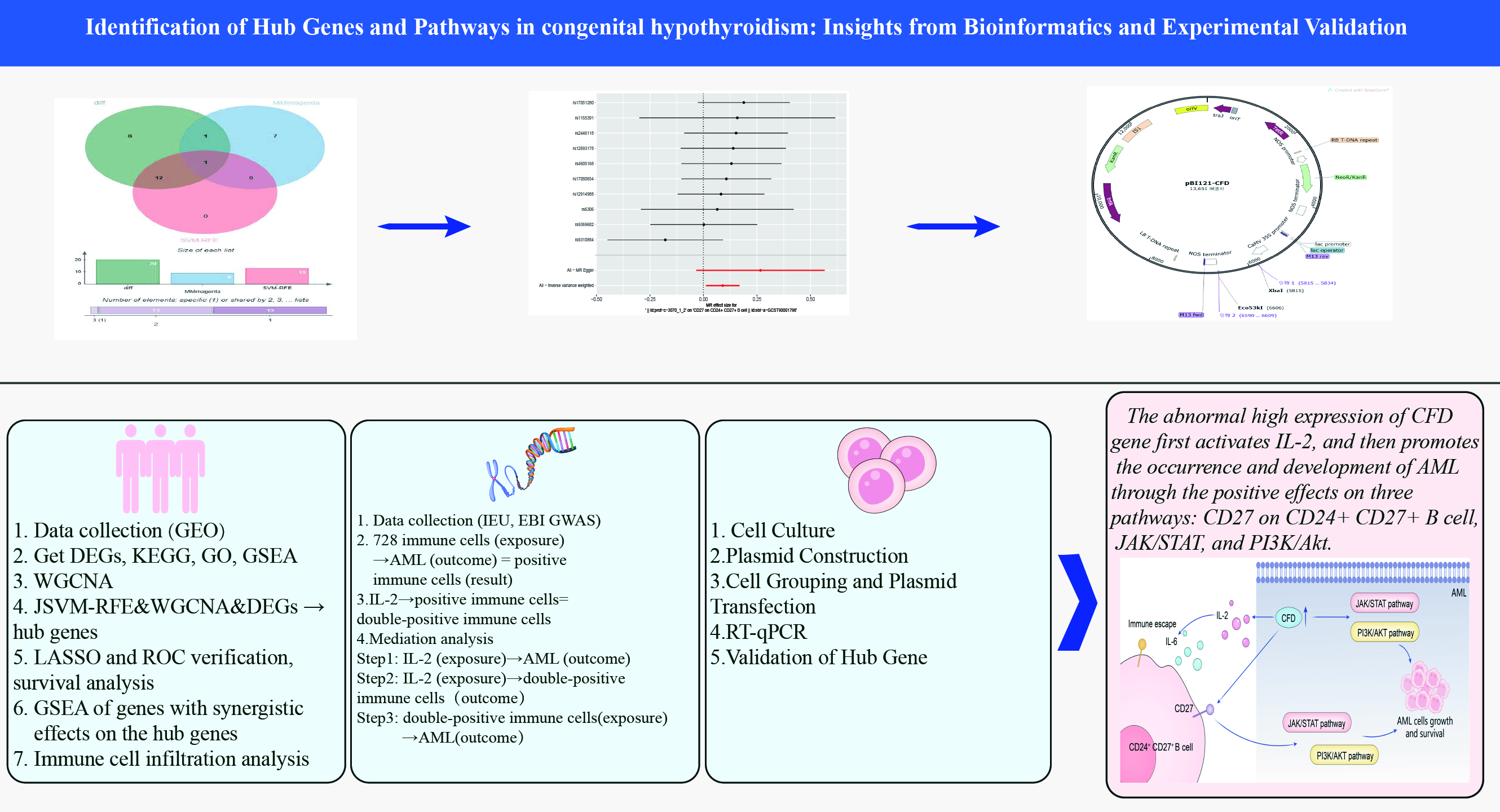

Supplement: Supplementary file 5 [file Image1.tif]
