# Supplementary material for: Identification of hub genes and immune-related pathways in acute myeloid leukemia: insights from bioinformatics and experimental validation
Source: Front Immunol. 2025 Jan 10;15:1511824. doi: 10.3389/fimmu.2024.1511824 (PMC11757261; doi:10.3389/fimmu.2024.1511824)
Supplement: Supplementary file 13 [file Table8.docx]

**STROBE-MR checklist of recommended items to address in reports of Mendelian randomization studies**^1^ ^2^

| **Item No.** | **Section** | **Checklist item** | **Page No.** | **Relevant text from manuscript** |
| --- | --- | --- | --- | --- |
| 1 | **TITLE and ABSTRACT** | Indicate Mendelian randomization (MR) as the study’s design in the title and/or the abstract if that is a main purpose of the study | 1 | Through Mendelian randomization(MR) analysis, on the one hand, we look for related immune cells, and on the other hand, we use it to determine the causal relationship among immune cells, immune mediators, and AML. |
|  | **INTRODUCTION** |  |  |  |
| 2 | **Background** | Explain the scientific background and rationale for the reported study. What is the exposure? Is a potential causal relationship between exposure and outcome plausible? Justify why MR is a helpful method to address the study question | N | Not performed |
| 3 | **Objectives** | State specific objectives clearly, including pre-specified causal hypotheses (if any). State that MR is a method that, under specific assumptions, intends to estimate causal effects | 2 | In this study, our objective is to first screen for hub genes and identify the inflammatory factors they act on through enrichment analysis. Subsequently, we utilize bulk MR to screen for immune cells and employ mediation MR to determine the relationship between inflammatory factors, immune cells, and AML. |
|  | **METHODS** |  |  |  |
| 4 | **Study design and data sources** | Present key elements of the study design early in the article. Consider including a table listing sources of data for all phases of the study. For each data source contributing to the analysis, describe the following: |  |  |
|  | a) | Setting: Describe the study design and the underlying population, if possible. Describe the setting, locations, and relevant dates, including periods of recruitment, exposure, follow-up, and data collection, when available. | 3 | The IEU database (https://gwas.mrcieu.ac.uk/) served as the source for this portion of the data. The IL-2 dataset (GWAS ID: prot-c-3070_1_2) include 501,428 SNPs from a European population. The EBI GWAS Catalog (https://www.ebi.ac.uk/gwas/) were source of the data on AML and immune cells, with the AML accession number GCST90435652. Immune cell data were collected under accession numbers GCST90274758 to GCST90274848, encompassing 728 immune cell types along with their corresponding GWAS IDs (Table S1), all derived from a European population. |
|  | b) | Participants: Give the eligibility criteria, and the sources and methods of selection of participants. Report the sample size, and whether any power or sample size calculations were carried out prior to the main analysis | N | Not performed |
|  | c) | Describe measurement, quality control and selection of genetic variants | 3 | Genome-wide significant SNPs with a threshold of P < 5×10−8 were included. In the absence of such SNPs, we considered those with P < 5×10−6 as potential instruments. We clustered SNPs based on linkage disequilibrium (window size = 10,000 kb and r² < 0.001), excluding weak instrumental variables (F-statistics < 10). |
|  | d) | For each exposure, outcome, and other relevant variables, describe methods of assessment and diagnostic criteria for diseases | 3-4 | We used inverse variance weighting (IVW) and MR-Egger methods as the primary methods for assessing causal relationships. Both methods needed to achieve a significance threshold of P < 0.05, and if neither method achieved this level, the IVW results were prioritized. IVW combines the causal effects represented by the Wald ratio of each SNP through meta-analysis, relying on the assumption that all SNPs are valid instruments. Therefore, this approach could be applied only after excluding SNPs exhibiting pleiotropy. |
|  | e) | Provide details of ethics committee approval and participant informed consent, if relevant | N | Not performed |
| 5 | **Assumptions** | Explicitly state the three core IV assumptions for the main analysis (relevance, independence and exclusion restriction) as well assumptions for any additional or sensitivity analysis | N | Not performed |
| 6 | **Statistical methods: main analysis** | Describe statistical methods and statistics used |  |  |
|  | a) | Describe how quantitative variables were handled in the analyses (i.e., scale, units, model) | N | Not performed |
|  | b) | Describe how genetic variants were handled in the analyses and, if applicable, how their weights were selected | N | Not performed |
|  | c) | Describe the MR estimator (e.g. two-stage least squares, Wald ratio) and related statistics. Detail the included covariates and, in case of two-sample MR, whether the same covariate set was used for adjustment in the two samples | N | Not performed |
|  | d) | Explain how missing data were addressed | 3 | Genome-wide significant SNPs with a threshold of P < 5×10−8 were included. In the absence of such SNPs, we considered those with P < 5×10−6 as potential instruments. |
|  | e) | If applicable, indicate how multiple testing was addressed | N | Not performed |
| 7 | **Assessment of assumptions** | Describe any methods or prior knowledge used to assess the assumptions or justify their validity | 7 | The results indicated that *CFD* gene is primarily associated with inflammation related to IL-2 and IL-6, as well as pathways involving PI3K/AKT and JAK/STAT3 signaling. |
| 8 | **Sensitivity analyses and additional analyses** | Describe any sensitivity analyses or additional analyses performed (e.g. comparison of effect estimates from different approaches, independent replication, bias analytic techniques, validation of instruments, simulations) | 4 | We assessed heterogeneity and horizontal pleiotropy by calculating P values. P > 0.05 indicated no significant heterogeneity or pleiotropy. Outliers were removed when detected, and causal estimates were recalculated. If significant heterogeneity persists following removal, a random effects model would be applied to assess result stability, as this model is less sensitive to weak SNP-exposure associations. We also conducted a leave-one-out analysis to evaluate the impact of each SNP on the overall causal estimate. |
| 9 | **Software and pre-registration** |  |  |  |
|  | a) | Name statistical software and package(s), including version and settings used | 6 | Statistical analyses were performed using SPSS 18.0 and R 4.1.1. P<0.05 was considered statistically significant. |
|  | b) | State whether the study protocol and details were pre-registered (as well as when and where) | N | Not performed |
|  | **RESULTS** |  |  |  |
| 10 | **Descriptive data** |  |  |  |
|  | a) | Report the numbers of individuals at each stage of included studies and reasons for exclusion. Consider use of a flow diagram | 7 | A Bulk MR analysis of 728 immune cell types identified 27 positive immune cells (Table S4).IL-2 has been determined to have a role in AML. |
|  | b) | Report summary statistics for phenotypic exposure(s), outcome(s), and other relevant variables (e.g. means, SDs, proportions) | 7 | Details regarding all exposure data SNPs involved in the above steps can be found in Table S5. Results of the five MR methods were presented in Table S6, and results of individual SNP analyses were detailed in Table S7. |
|  | c) | If the data sources include meta-analyses of previous studies, provide the assessments of heterogeneity across these studies | N | Not performed |
|  | d) | For two-sample MR:  i.  Provide justification of the similarity of the genetic variant-exposure associations between the exposure and outcome samples  ii.  Provide information on the number of individuals who overlap between the exposure and outcome studies | N | Not performed |
| 11 | **Main results** |  |  |  |
|  | a) | Report the associations between genetic variant and exposure, and between genetic variant and outcome, preferably on an interpretable scale | 7 | Scatter plots and corresponding odds ratios (OR) suggest that IL-2 influences AML by acting on CD27 on CD24+ CD27+ B cells (Figures 5K-5O). |
|  | b) | Report MR estimates of the relationship between exposure and outcome, and the measures of uncertainty from the MR analysis, on an interpretable scale, such as odds ratio or relative risk per SD difference | 7 | The role of IL-2 in AML is a promoting effect (Figures 5A, 5G). The overall effect estimate (beta_all) is 0.052, indicating no significant heterogeneity (Mendelian randomization Egger test method, P = 0.317; inverse variance weighted method, P = 0.385) or pleiotropy (P = 0.632).IL-2 exhibited a positive correlation with the dual positive immune cell(Figures 5B, 5H), with a beta estimate (beta1) of 0.090 (IVW method, OR=1.094; [95% CI, 1.011-1.183], P=0.025), and no significant heterogeneity (MR Egger method, P=0.827; IVW method, P=0.764) or pleiotropy (P=0.267). The dual positive immune cell was also positively associated with AML(Figures 5C, 5I), yielding a beta estimate (beta2) of 0.160 (IVW method, OR=1.173; [95% CI, 1.012-1.360], P=0.034), and no significant heterogeneity (MR Egger method, P=0.665; IVW method, P=0.685) or pleiotropy (P=0.462) was observed. |
|  | c) | If relevant, consider translating estimates of relative risk into absolute risk for a meaningful time period | N | Not performed |
|  | d) | Consider plots to visualize results (e.g. forest plot, scatterplot of associations between genetic variants and outcome versus between genetic variants and exposure) | 7 | Further calculations revealed a mediating effect of 0.0144 (beta12 = beta1 x beta2) and a direct effect of 0.0376 (beta_dir = beta_all - beta12), with the mediating effect contributing to 27.69% of the total effect (beta12_p = beta12 / beta_all). The funnel plots displayed a symmetric distribution without apparent outliers, suggesting minimal heterogeneity (Figures 5D-5F). The leave-one-out forest plots indicated the robustness of the results, with no significant outliers detected (Figures 5J-5L). |
| 12 | **Assessment of assumptions** |  |  |  |
|  | a) | Report the assessment of the validity of the assumptions | N | Not performed |
|  | b) | Report any additional statistics (e.g., assessments of heterogeneity across genetic variants, such as *I^2^*, Q statistic or E-value) | N | Not performed |
| 13 | **Sensitivity analyses and additional analyses** |  |  |  |
|  | a) | Report any sensitivity analyses to assess the robustness of the main results to violations of the assumptions | 7 | The funnel plots displayed a symmetric distribution without apparent outliers, suggesting minimal heterogeneity (Figures 5D-5F). The leave-one-out forest plots indicated the robustness of the results, with no significant outliers detected (Figures 5J-5L). |
|  | b) | Report results from other sensitivity analyses or additional analyses | N | Not performed |
|  | c) | Report any assessment of direction of causal relationship (e.g., bidirectional MR) | 7 | IL-2 has been determined to have a role in AML. |
|  | d) | When relevant, report and compare with estimates from non-MR analyses | N | Not performed |
|  | e) | Consider additional plots to visualize results (e.g., leave-one-out analyses) | 7 | The leave-one-out forest plots indicated the robustness of the results, with no significant outliers detected (Figures 5J-5L). |
|  | **DISCUSSION** |  |  |  |
| 14 | **Key results** | Summarize key results with reference to study objectives | 7 | Scatter plots and corresponding odds ratios (OR) suggest that IL-2 influences AML by acting on CD27 on CD24+ CD27+ B cells. |
| 15 | **Limitations** | Discuss limitations of the study, taking into account the validity of the IV assumptions, other sources of potential bias, and imprecision. Discuss both direction and magnitude of any potential bias and any efforts to address them | 9 | Our study still has certain limitations. First, the results of the MR analysis have not been experimentally verified. Second, the MR data is from the European population and lacks data from other populations. |
| 16 | **Interpretation** |  |  |  |
|  | a) | Meaning: Give a cautious overall interpretation of results in the context of their limitations and in comparison with other studies | 8 | Our subsequent in-depth research shows that *CFD* gene ultimately promotes the progression of AML by activating IL-2 and then activating CD27 on CD24+ CD27+ B cells.IL-2 can regulate B cells, which is consistent with previous studies(37,38). |
|  | b) | Mechanism: Discuss underlying biological mechanisms that could drive a potential causal relationship between the investigated exposure and the outcome, and whether the gene-environment equivalence assumption is reasonable. Use causal language carefully, clarifying that IV estimates may provide causal effects only under certain assumptions | N | Not performed |
|  | c) | Clinical relevance: Discuss whether the results have clinical or public policy relevance, and to what extent they inform effect sizes of possible interventions | 9 | Our study has confirmed the impact of the three subsequent immune pathways dominated by *CFD* gene and mediated by IL-2 on AML. This is undoubtedly of great significance for enriching the immune theory related to AML and improving its related immunotherapy. |
| 17 | **Generalizability** | Discuss the generalizability of the study results (a) to other populations, (b) across other exposure periods/timings, and (c) across other levels of exposure | N | Not performed |
|  | **OTHER INFORMATION** |  |  |  |
| 18 | **Funding** | Describe sources of funding and the role of funders in the present study and, if applicable, sources of funding for the databases and original study or studies on which the present study is based | 10 | This work was supported by the Shandong Maternal and Child Health Association for their support through grant No. SFYXH-2023W043.  We thank Weifang City young medical talent lifting project support. |
| 19 | **Data and data sharing** | Provide the data used to perform all analyses or report where and how the data can be accessed, and reference these sources in the article. Provide the statistical code needed to reproduce the results in the article, or report whether the code is publicly accessible and if so, where | 9 | All data will be made available upon request to the corresponding author. |
| 20 | **Conflicts of Interest** | All authors should declare all potential conflicts of interest | 9 | The authors declare that the research was conducted in the absence of any commercial or financial relationships. |

This checklist is copyrighted by the Equator Network under the Creative Commons Attribution 3.0 Unported (CC BY 3.0) license.

1. Skrivankova VW, Richmond RC, Woolf BAR, Yarmolinsky J, Davies NM, Swanson SA, et al. Strengthening the Reporting of Observational Studies in Epidemiology using Mendelian Randomization (STROBE-MR) Statement. JAMA. 2021;under review.

2. Skrivankova VW, Richmond RC, Woolf BAR, Davies NM, Swanson SA, VanderWeele TJ, et al. Strengthening the Reporting of Observational Studies in Epidemiology using Mendelian Randomisation (STROBE-MR): Explanation and Elaboration. BMJ. 2021;375:n2233.
